# A 3D spatial proteomic map of the human pancreatic islet microenvironment

**DOI:** 10.21203/rs.3.rs-10144543/v1

**Published:** 2026-07-10

**Authors:** Yumi Kwon, Shane S. Kelly, Jing Chen, James Carson, Lye Meng Markillie, Hu Dehong, Geremy Clair, James Labyer, Demi Awosika-Olumo, Erik Ferlanti, Ronald J. Moore, Clayton E. Mathews, Martha Campbell-Thompson, Ying Zhu, Ernesto S. Nakayasu, Wei-Jun Qian

**Affiliations:** 1 Environmental Molecular Sciences Division, Pacific Northwest National Laboratory, Richland, WA.; 2 Biological Sciences Division, Pacific Northwest National Laboratory, Richland, WA.; 3 Department of Pathology, Immunology and Laboratory Medicine, College of Medicine, University of Florida, Gainesville, FL.; 4 The University of Texas at Austin, Texas Advanced Computing Center, Austin, TX; 5 Department of Infectious Disease and Immunology, College of Veterinary Medicine, University of Florida, Gainesville, FL; 6 Present address: Department of Proteomic and Genomic Technologies, Genentech, South San Francisco, CA.

## Abstract

The human pancreas is a structurally and functionally complex organ in which endocrine islets are embedded within an exocrine matrix. Despite advances in spatial omics, three-dimensional (3D) proteomic map of the human islet microenvironment remains lacking. Here, we present a 3D spatial proteomics workflow that integrates immunofluorescence imaging, laser capture microdissection, nanoPOTS processing, and LC-MS/MS to map the islet microenvironment at 50 μm resolution, achieving ~3,000 protein identifications with spatial fidelity. Unsupervised clustering analyses revealed four molecularly distinct microenvironments spanning the acinar-to-islet axis, including a previously underappreciated peri-islet ductal-stromal niche enriched with canonical collagens and other extracellular matrix (ECM) proteins. Spatial correlation analysis (linking abundances to relative distance from the islet center) revealed proteins with interesting, reversed correlation patterns between islet and acinar compartments, which were enriched with ECM and cytoskeletal components. Finally, we provide a publicly accessible interactive web-based platform, enabling integrated 3D visualization of the spatial proteome alongside immunofluorescence imaging. Collectively, this work establishes a proof-of-principle framework for studying spatial tissue proteomic profiles, advances our understanding of the islet microenvironment, and lays a potential foundation for future applications in disease research.

## Introduction

The pancreas plays a central role in regulating the body’s glucose homeostasis and metabolism through its endocrine function and in supporting digestion system by exocrine enzyme secretion^1^. The endocrine compartment, the islets of Langerhans, consists of specialized micro-organs embedded within the exocrine pancreas. Although islets represent only ~2% of pancreatic mass, they secrete key hormones, including insulin from β-cells, glucagon from α-cells, somatostatin from δ-cells, pancreatic polypeptide from PP cells, and ghrelin from ε-cells^2^. These hormones function in a tightly coordinated network to maintain energy balance, particularly blood glucose levels. The exocrine pancreas is primarily composed of acinar and ductal cells, which secrete digestive enzymes into duodenum to break down dietary macromolecules for intestinal absorption^1^. Crosstalk between endocrine and exocrine compartments is essential for both pancreatic development and functional coordination^3^.

Human islet microenvironments are organized within a complex anatomical framework, in which endocrine cell clusters are embedded in exocrine cells, vasculature, nerves, stromal cells, and layered extracellular matrix (ECM). Perturbations in islet microenvironments, including ECM composition, are increasingly recognized as contributors to β-cell dysfunction and diabetes^4, 5^, highlighting the need for spatially resolved molecular maps of the human islet niche. However, three-dimensional (3D) molecular maps of human islets remain poorly defined, limiting mechanistic understanding of how local cellular or cell–matrix interactions support islet function. These challenges are addressed by the Human BioMolecular Atlas Program (HuBMAP)^6^, a consortium effort to generate comprehensive, spatially resolved molecular atlases of human tissues. Within this framework, high-resolution, proteome-level characterization of three-dimensional islet microenvironments remains limited.

Recent advances in spatial transcriptomics, including application to human pancreas and islets^7^, are highly promising, but mRNA often displays poor correlation with protein abundance^8^. Spatial transcriptomics is inherently blind to post-translational modifications and provides only limited information on functional states, protein-protein interactions, secreted proteins, and the ECM. Spatial proteomics can directly quantify protein levels, localization, and ECM components, enabling inference of signaling interactions, functional interactions, and cell–ECM relationships that transcriptome alone cannot resolve. Antibody-based imaging methods, including cyclic immunofluorescence (cycIF)^9^, co-detection by indexing (CODEX)^10^, multiplexed ion beam imaging (MIBI)^11^, iterative bleaching extends multiplexity (IBEX)^12^, and imaging mass cytometry (IMC)^13, 14, 15^, enable high-resolution visualization of dozens to hundreds of proteins within tissue sections, but are constrained by antibody availability and multiplexing limits (~250 proteins to date^16, 17^). In contrast, mass spectrometry (MS)-based spatial proteomics allows unbiased and deep proteome coverage across multiple cell types and ECM components^18, 19, 20^.

In pancreas, IMC has been applied to study human islets during type 1 diabetes (T1D) progression^21^, revealing changes in islet architecture, a progressive loss of β-cell markers, and immune cell recruitment^22, 23^. Previously, we demonstrated the feasibility of 2D nanoPOTS-based spatial proteomics, mapping ~3,500 proteins across the islet and adjacent acinar regions^24^. However, 2D mapping cannot resolve the full volumetric structure of a single islet microenvironment, obscure protein gradients along the z-axis. To address this, we developed a 3D nanoPOTS-based workflow integrating immunofluorescence (IF) imaging, laser capture microdissection (LCM) of serial sections, and advanced nanoLC-MS/MS analysis to reconstruct a volumetric spatial proteomic atlas of a single human pancreatic islet microenvironment. By combining spatial proteomics with multiplex IF imaging, we captured the spatial complexity of islets and their interactions with adjacent acinar and ductal regions. This approach reveals molecular gradients and cell-type–specific protein distributions across the islet, acinar, and ductal compartments, providing a high-content reference for the normal human islet niche and establishing a potential framework for future studies of islet remodeling in diabetes and other pancreatic diseases.

## Results

### 3D spatial proteomic mapping applying the LCM-nanoPOTS workflow.

**Fig. 1 |:**
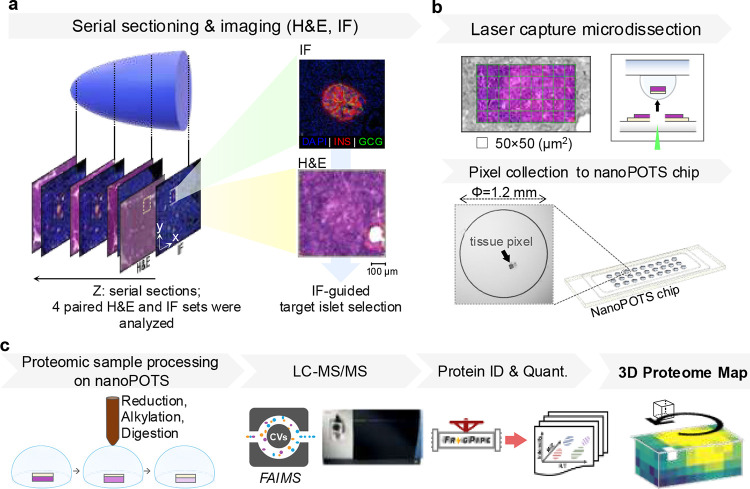
Workflow of 3D spatial proteomics of a human islet. **a** Schematic illustration of the 3D spatial proteomics workflow. Serial tissue sectioning and imaging were performed using H&E staining and immunofluorescence (IF) markers (GCG, INS, DAPI), allowing identification of the islet across multiple z-layers at 40 μm intervals and target islet selection for downstream analysis. **b** Target islet and its adjacent area on H&E-stained sections were pixelated and dissected using laser capture microdissection (LCM). Dissected and catapulted tissue pixels were directly captured on the nanoPOTS **c** following sample collection on the nanoPOTS chip, each pixel underwent processing on the nanoPOTS platform, including reduction, alkylation, and digestion. The resulting peptides were analyzed using LC-FAIMS-MS/MS. Protein identification and quantification were performed using FragPipe, enabling 3D proteome mapping to resolve the spatial and molecular composition of the islet.

Our 3D spatial proteomic mapping approach utilizes a serial-section, LCM-nanoPOTS workflow that links 3D islet anatomy to pixel-level protein profiles (Figure 1). Serial 10-μm sections were generated from a carboxymethylcellulose (CMC)-embedded pancreatic tissue block of an adult pancreas donor^25, 26^. Four paired sets of Hematoxylin and Eosin (H&E)-stained and immunofluorescence (IF)-stained sections, spaced at 40-μm intervals along the z-axis, spanning the widest region of the islet towards the end, were selected to capture its 3D structure of the islet. For each pair, one section was stained by IF for insulin (INS) and glucagon (GCG) together with the nuclear marker DAPI, allowing identification of islets across multiple z-layers. Guided by these IF images, a target islet was identified and selected for downstream analysis on the immediate adjacent H&E-stained PEN membrane slide (Figure 1a, **Supplementary Table 1**).

The target islet and the peri-islet area on each H&E-stained section were then pixelated into a 50 × 50 μm^2^ grid and dissected using laser capture microdissection (LCM). Dissected and catapulted tissue pixels were directly captured onto a nanoPOTS chip, enabling low-loss proteomic processing at nanoscale volumes^24, 27, 28, 29^ (Figure 1b, **Supplementary Table 2**). Following sample collection on the nanoPOTS chip, each pixel underwent reduction, alkylation, and tryptic digestion on the nanoPOTS platform. The resulting peptides were analyzed by LC-MS/MS on an Orbitrap Lumos Tribrid mass spectrometer equipped with a FAIMS interface. To enhance the proteomic coverage from the small tissue pixels, we employed the TIFF (transferring identification based FAIMS filtering) strategy, leveraging FAIMS-CV–resolved spectral libraries for peptide identifications^24, 30^. Protein identification and label-free quantification were performed using FragPipe. The overall LCM-nanoPOTS-LC-MS/MS workflow enables 3D proteome mapping that resolves the spatial and molecular composition of the islet and its surrounding peri-islet regions across the sampled sections (Figure 1c).

**Fig. 2 |:**
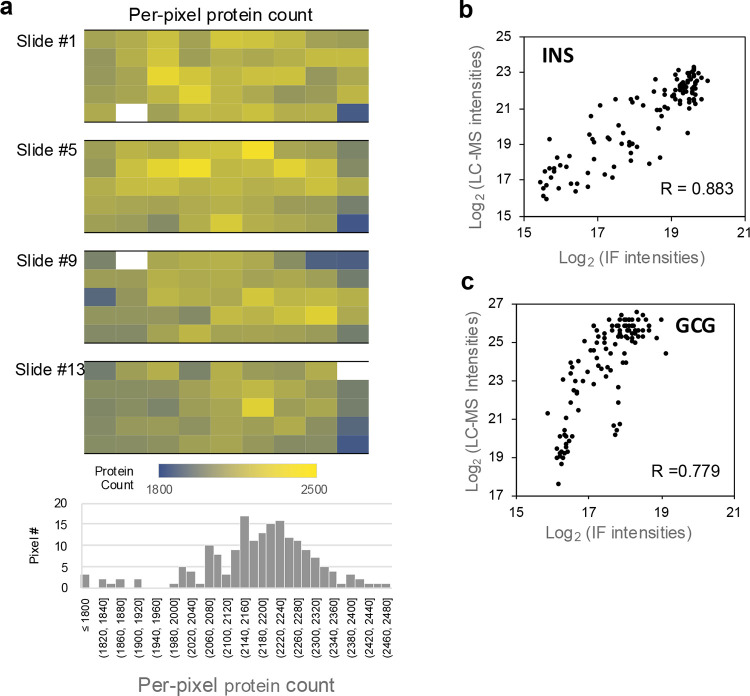
Comprehensive Analysis of Spatial Proteomic Data **a** Spatial heatmaps showing the number of quantified proteins per pixel across serial pancreatic tissue sections, with the histogram summarizing the distribution of per-pixel protein counts. **(b-c)** Scatter plots showing the correlation between intensities from immunofluorescence staining and proteomics data for insulin (INS, b) and glucagon (GCG, c).

Across the 177 successfully dissected pixels out of the intended 180 (9 columns × 5 rows × 4 sections), we quantified 3,187 proteins in total, with an average of 2,159 proteins identified per pixel (**Supplementary Table 3)**. Three pixels were lost due to technical failures during microdissection. The spatial distribution of per-pixel protein counts across the four tissue sections (Slides #1, #5, #9, and #13) is shown alongside a histogram summarizing the overall distribution of quantified proteomes (Figure 2a). To assess the accuracy of label-free quantification, we compared LC-MS-derived protein intensities for INS and GCG with their corresponding IF intensities on adjacent sections (**Supplementary Table 4)**. This analysis revealed strong Pearson correlations for both markers (INS, r = 0.883, Figure 2b; GCG, r = 0.779, Figure 2c), confirming the robustness of the mass spectrometry-based measurements. Notably, LC-MS/MS provided a wider and more linear dynamic range of protein intensities for INS and GCG than IF, highlighting the advantages of MS-based label-free quantification for accurately capturing protein abundances across a broad range of abundance levels. Collectively, these data demonstrate deep, quantitative spatial proteome coverage of the human pancreatic islet microenvironment.

### 3D spatial context of the islet microenvironment

**Fig. 3 |:**
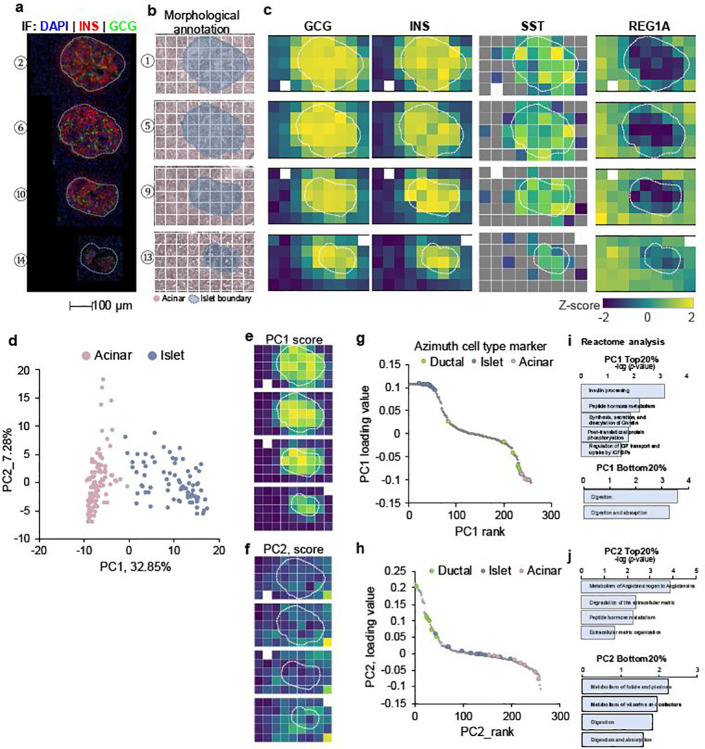
3D proteome mapping and unsupervised proteome analysis of a single Islet **a** Immunofluorescence staining shows a single islet across four consecutive sections. **b** H&E-stained sections, used for LCM proteomics, are annotated as acinar or islet based on morphological features. **c** Spatial proteome mapping illustrates well-known markers: Glucagon (GCG, α-cell marker), Insulin (INS, β-cell marker), Somatostatin (SST, δ-cell marker), and regenerating islet-derived protein 1 alpha (Reg1A, acinar marker). **d** PCA plot illustrates clear separation between islet and acinar pixels, with PC1 accounting for 32.85% of the variance. **e-f** Mapping of PC1 and PC2 scores on the tissue grid shows elevated PC1 scores in islet centers and reduced scores in acinar regions. PC2 reveals high scores in the bottom-right corner, with extended H&E views confirming proximity to ductal cells (Supplementary Figure 1). **g-h** Ranking of loading scores for PC1 and PC2 indicates strong overlap with Azimuth islet, acinar, and ductal cell markers, consistent with single-cell RNA sequencing data. **i-j** Functional enrichment analysis of the PC1 top/bottom 20% score-driving proteins: PC1 positive scores are enriched in hormonal metabolism, negative scores in digestion; PC2 top 20% proteins are enriched in ECM organization and hormone metabolism.

To investigate the spatial organization of the islet microenvironment at the proteome level, we compared pixel-level protein maps with H&E-images and IF-images. High-resolution IF images of adjacent sections visualized INS and GCG along with DAPI (Figure 3a), allowing precise delineation of islet boundaries. On the corresponding H&E-stained sections from which tissue pixels were isolated by LCM, each pixel was manually annotated as islet or acinar based on morphological features, with islet boundaries and acinar regions explicitly demarcated across four corresponding H&E- images of the adjacent sections (Figure 3b, **Supplementary Table 5**). Mapping z-scored protein abundances back onto their original pixel locations produced spatial proteome heatmaps that recapitulate expected endocrine-exocrine patterns (Figure 3c). Endocrine markers, including GCG (α cells), INS (β cells), and somatostatin (SST, δ cells), exhibited strong enrichment within the islet contour, whereas the exocrine marker regenerating islet-derived protein 1α (REG1A) was predominantly localized to surrounding acinar areas. Because the sampled region represents a half-sphere portion of an islet, the islet cross-sectional area decreased from the top to bottom sections as reflected in the proteome maps.

To further explore the spatial context captured by our proteomic data, we performed principal component analysis (PCA) to test whether unsupervised analysis can delineate acinar vs. islet tissue. The first principal component (PC1), accounting for 32.85% of the variance, clearly separated pixels corresponding to islet- and acinar-dominant regions (Figure 3d). Notably, pixels located centrally within the islet exhibited higher PC1 scores, while those in acinar regions displayed lower PC1 scores. Spatially mapping PC1 scores further confirmed this separation, correlating pixel locations with their proteomic profiles (Figure 3e). Interestingly, PC2 scores (7.28% of variance) highlighted localized molecular features, particularly in bottom-right corner pixels of each section (Figure 3f). Careful reexamination of the tissue found that these pixels were located near a duct (**Supplementary Figure 1**), linking ductal regions to distinct proteomic signatures.

To uncover proteins driving variance in PC1 and PC2, we ranked the principal component loading scores of each protein and compared them to curated list of pancreatic cell-type markers from single-cell RNA sequencing (scRNAseq) datasets from Azimuth (Figure 3g, h). In agreement with the spatial data, PC1 loading scores were driven by islet cell markers (positive score) and acinar cell markers (negative scores), while PC2 loading scores were primarily driven by ductal cell markers (positive scores).

To better understand the function of the score-driving proteins, we performed Reactome pathway enrichment analysis on the top and bottom 20% of loading scores for each component. The top 20% of PC1 positive loading scores, enriched in islets markers, were associated with insulin processing, peptide hormone metabolism, protein phosphorylation, and regulation of IGF transport and uptake by IGFBPs (Figure 3i). The bottom 20% of PC1 negative loading scores, enriched with acinar markers, were associated with digestion and digestion & absorption pathways (Figure 3i). The top 20% of PC2 positive loading scores, enriched with ductal markers, were associated with metabolism of angiotensinogen to angiotensins, degradation of the extracellular matrix, peptide hormone metabolism, and extracellular matrix organization (Figure 3j). The bottom 20% of PC2 loading scores were associated with metabolism of folate and pterines, metabolism of vitamins and cofactors, digestion, and digestion and absorption (Figure 3j).

The proteins with the highest and lowest loadings for PC1 and PC2 demonstrate distinct spatial patterns, reflecting their potential functional roles in pancreatic tissue organization and physiology (**Supplementary Figure 2**). CRMP1, the top contributing protein for PC1, localized to islet regions, reflecting its role in signaling and morphology regulation, whereas IQGAP2, the lowest PC1 contributor, was enriched in acinar compartments, consistent with its function in cytoskeletal dynamics. For PC2, COL3A1, the top contributor, was associated with ductal regions, highlighting its role in extracellular matrix organization, while ENPP1, the lowest contributor, localized to peripheral acinar areas, aligning with its role in nucleotide metabolism. These results demonstrate that unsupervised analysis effectively captures tissue-specific proteomic signatures, establishing clear links between PCA-driven patterns and tissue-specific spatial localization, and supporting the spatial fidelity and biological relevance of the dataset.

### Molecular characterization of spatial clusters

**Fig. 4 |:**
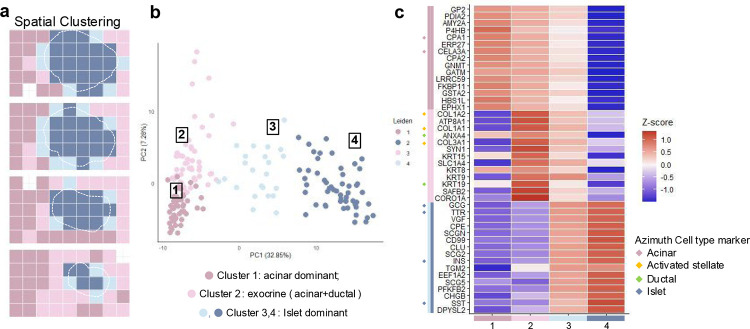
Spatial clustering of proteomic data reveals expected patterns in pancreatic tissue. **a** Grid mapping of pancreatic tissue pixels colored using the four Leiden clusters (Cluster 1–4), representing distinct molecular profiles identified through unsupervised clustering of spatial proteomic data. Cluster 1 predominantly corresponds to acinar regions, Cluster 2 highlights duct-adjacent transitional regions, while Cluster 3 and 4 delineate distinct islet microenvironments, with Cluster 3 capturing boundary pixels and Cluster 4 representing the islet core. **b** PCA plot from Figure 3 with color-coded by Leiden cluster assignment, revealing the alignment of clusters with PC1, which reflects the molecular continuum from exocrine regions (Cluster 1 and 2) to endocrine regions (Cluster 3 and 4). **c** Heatmap showing Z-score-normalized abundance of the top significantly enriched proteins identified by scran one-versus-all differential analysis for each Leiden cluster. Clusters 3 and 4 are displayed together because their top differentially enriched protein profiles showed substantial overlap. Colored side markers indicate proteins that overlap with Azimuth pancreas cell type markers, including acinar, activated stellate, ductal, and islet markers.

To move beyond binary islet/acinar classification, we employed unsupervised clustering using the Leiden algorithm. The Leiden algorithm is a graph-based community detection method designed to optimize clustering by iteratively maximizing modularity—a measure of intra-cluster connection density relative to inter-cluster connections^31^. This approach is well-suited for identifying proteomic patterns in high-dimensional datasets without relying on pre-assigned spatial information^32, 33, 34^. This method enabled the classification of all 177 tissue pixels based on their proteomic profiles, using the most variable proteins across the dataset. By focusing exclusively on molecular features, the analysis identified four distinct Leiden clusters (Figure 4a, **Supplementary Table 5**). These clusters exhibited unique proteomic signatures that aligned closely with histological features observed in adjacent tissue sections stained for IF and H&E (Figure 3a, b). Cluster 1 primarily comprised pixels from acinar regions, with a molecular signature dominated by exocrine-related proteins (**Supplementary Figure 3a, b**). Cluster 2 contained a mixture of acinar pixels and those located near ductal structures (**Supplementary Figure 3c, d**). This distinction corresponded with high PC2 scores observed in earlier PCA analysis (Figure 3f), suggesting that Cluster 2 represents a transitional molecular profile shaped by proximity to ducts and mixed cell types. Clusters 3 and 4 were localized within islet regions and captured distinct aspects of the islet microenvironment. While these clusters displayed similar metagene patterns (**Supplementary Figure 3 e-g**), closer examination of protein intensities revealed meaningful differences. Cluster 3 encompassed boundary pixels at the interface between islet and acinar regions, characterized by blended proteomic signatures indicative of transitional molecular environments. Cluster 4, by contrast, was confined to the islet core, with a proteomic profile reflecting the central islet cells. Mapping the Leiden clusters onto PCA space revealed their alignment with PC1, illustrating a continuum from exocrine to endocrine profiles (Figure 4b).

To characterize molecular differences between clusters, we performed one-versus-all differential analysis using the scran method^35^ and selected the top 15 significantly enriched proteins for each Leiden cluster. The heatmap shows the Z-score normalized abundances of these proteins (Figure 4c). To provide cell type context for these scran-derived markers, we compared the identified proteins with Azimuth pancreas cell type markers^36, 37, 38, 39, 40^. Proteins overlapping with these reference markers are indicated in Figure 4c, and the complete list of Azimuth markers detected in this study is summarized in **Supplementary Table 6**. The resulting heatmap revealed a clear molecular transition from acinar-dominant (Cluster 1), exocrine/ductal (Cluster 2), to islet-dominant regions (Clusters 3 and 4), with distinct protein signatures that not only confirmed the expected endocrine–exocrine partitioning but also uncovered previously underappreciated molecular features of the peri-islet microenvironment.

Cluster 1 markers were dominated by classical acinar enzymes and secretory proteins, including GP2, AMY2A, CPA1, CPA2, and CELA3A, confirming its strong exocrine identity^1^. Many of these proteins are central to digestive enzyme production and secretion, a hallmark of acinar cell function. In addition, proteins such as ER-resident folding factors (e.g., PDIA2, P4HB, ERP27, FKBP11) and metabolic enzymes (e.g., GATM, GSTA2) further support the high secretory and biosynthetic activity characteristic of acinar cells^1^. Notably, GP2, whose uromodulin-like domain architecture suggests potential fibrillar scaffold activity, is an intriguing acinar-derived matrix-like protein warranting further investigation.

Cluster 2 markers exhibited a distinct enrichment of proteins associated with extracellular matrix (ECM) organization and cytoskeletal structure, including fibrillar collagens (COL1A1, COL1A2, COL3A1) alongside ductal-associated proteins (KRT8, KRT15, KRT19, and SLC14A)^36, 39^. Our analysis suggests that Cluster 2 represents more than a simple ductal compartment, but rather a peri-islet ductal–stromal transition zone enriched in ECM and matrix-adjacent proteins. Beyond the canonical collagens, Cluster 2 uniquely co-enriched several proteins with underappreciated roles in ECM biology, including ANXA4, SAFB2, ATP8A1, and CORO1A. ANXA4, traditionally viewed as a membrane-associated Ca^2+^-binding protein, has been increasingly implicated in cell matrix adhesion and basement membrane organization, positioning it as a candidate ductal-ECM interface protein^41, 42^. The co-enrichment of keratins with fibrillar collagens is consistent with recent reports of extracellular keratin fragments acting as matricellular signals^43, 44^.

Clusters 3 and 4 were both enriched for endocrine-associated proteins, with substantial overlap consistent with their shared islet localization. Both clusters were characterized by strong enrichment of canonical islet hormones and secretory granule proteins, including GCG (alpha cells), INS (beta cells), SST (delta cells), CHGB, SCG5, and CPE, reflecting active hormone production and regulated secretion. These proteins are central to endocrine function, including prohormone processing (CPE), granule formation (CHGB, SCG5), and peptide hormone release^36, 38, 45, 46^.

Consistent with their shared identity, cluster–cluster correlation analysis revealed a near-perfect Pearson correlation between Clusters 3 and 4 (**Supplementary Figure 4a**). However, the heatmap revealed a clear core-to-mantle proteomic gradient within the islet compartment. Cluster 4 exhibited relatively strongest enrichment of hormone-processing and secretion-associated proteins, suggesting a more functionally islet core phenotype (**Supplementary Figure 4b)**. In contrast, Cluster 3 displayed intermediate enrichment of these proteins and partial overlap with non-endocrine features, consistent with an islet mantle/peri-islet boundary region transitioning into surrounding exocrine tissue.

Importantly, the islet-dominant clusters also revealed proteins with potential roles as novel islet-associated ECM modulators. TGM2 (transglutaminase 2), a well-established extracellular crosslinker of fibronectin and collagen, was enriched in Clusters 3 and 4—an interesting observation given TGM2’s underexplored role in islet matrix remodeling and its potential relevance to islet fibrosis in type 2 diabetes^47, 48, 49^. Similarly, CLU (clusterin), an increasingly recognized matricellular protein that modulates ECM aggregation and TGF-β signaling, was elevated in islet-dominant regions, suggesting an islet-intrinsic mechanism for fine-tuning the local matrix environment. Additional islet-enriched proteins—including VGF, SCGN, TTR, PFKFB2, EEF1A2, and DPYSL2—further expand the islet proteomic signature beyond canonical hormones, pointing to coordinated neurosecretory and metabolic programs within the islet core^36, 38, 45^.

### 3D Spatial proteomic profiles reveal abundance gradients of the islet microenvironment

**Fig. 5 |:**
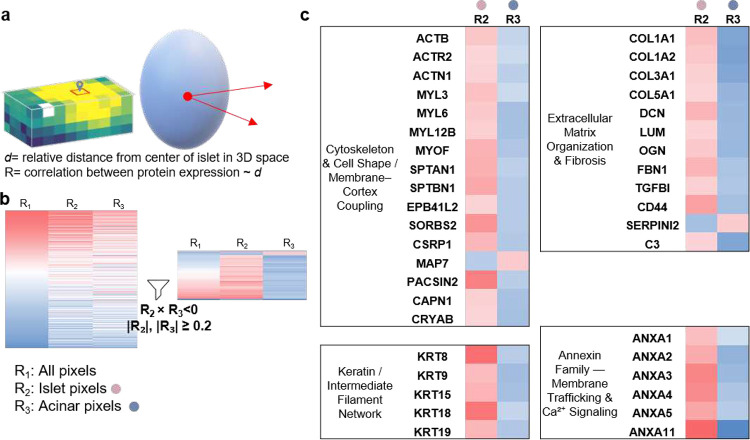
Correlation analysis of protein intensities with distance from islet core in islet. **a** Illustration of distance calculation (*d*) from the islet center to each pixel using 3D spatial coordinates, linking this spatial context to protein abundance levels (R). **b** Heatmap of correlations across all pixels (R1), islet pixels (R2), and acinar pixels (R3), highlighting proteins with reversed patterns in islet and acinar regions. **c** Selected proteins were grouped using Gene Ontology Biological Process and Cellular Component annotations and visualized as heatmaps of R2 and R3. The major groups include cytoskeleton and membrane–cortex coupling, extracellular matrix organization and fibrosis, keratin/intermediate filament network, and annexin-mediated membrane trafficking and Ca^2+^ signaling. Pink and blue indicate positive and negative correlations with distance, respectively.

To investigate spatial protein abundance gradients in pancreatic tissue, we examined the relationship between protein abundances and their spatial location relative to the islet center through correlation analysis. Using the x, y, and z coordinates of each pixel, we computed the relative distance (*d*) from the geometric center of the islet defined based on the overall islet morphology and linked the spatial context to protein abundance levels (Figure 5a, **Supplementary Table 7**). This framework enabled us to explore how protein abundance gradients align with the islet core, revealing potential abundance or concentration patterns across the endocrine-exocrine axis. We next calculated correlation coefficients for each protein across three spatial contexts: all pixels (R_1_), islet pixels only (R_2_), and acinar pixels only (R_3_) (Figure 5b). This stratified correlation framework allowed us to distinguish proteins displaying global gradients across the tissue from those showing compartment-specific spatial behavior. To focus on proteins with divergent patterns between endocrine and exocrine regions, we selected candidates with opposite correlation directions between islet and acinar pixels (R_2_ × R_3_ < 0), requiring both |R_2_| and |R_3_| to be at least 0.2. This filtering strategy isolated proteins whose spatial abundance gradients were reversed between the two pancreatic microenvironments, suggesting that the same molecular machinery may adopt distinct spatial organization patterns depending on tissue context.

Functional annotation of the selected proteins using Gene Ontology Biological Process and Cellular Component terms revealed four predominant functional groups (Figure 5c): Proteins in ‘extracellular matrix organization and fibrosis’ include fibrillar collagens (COL1A1, COL1A2, COL3A1, COL5A1), small leucine-rich proteoglycans (DCN, LUM, OGN), microfibrillar components (FBN1), matricellular signaling proteins (TGFBI, CD44), the pancreas-enriched protease inhibitor SERPINI2, and the complement component C3—exhibited reversed correlation directions between islet and acinar compartments. This pattern is consistent with spatially restructured stromal and ECM environments across the islet–exocrine interface, in line with the peri-islet ductal–stromal niche identified by our Leiden clustering analysis and reinforcing the notion of active ECM remodeling at the endocrine–exocrine boundary.

Cytoskeletal and membrane–cortex-associated proteins include actin and actin regulators (ACTB, ACTR2, ACTN1), myosin light chains (MYL3, MYL6, MYL12B), the muscle-like ferlin MYOF, spectrin cytoskeleton components (SPTAN1, SPTBN1, EPB41L2), adapter and remodeling proteins (SORBS2, PACSIN2, CAPN1), and the chaperone CRYAB. A distinct group of Keratin and intermediate filament proteins composed of KRT8, KRT9, KRT15, KRT18, and KRT19 showed spatially divergent abundance patterns between islet and acinar regions. These proteins may reflect differences in epithelial structural organization or local tissue architecture along the endocrine–exocrine boundary.

A particularly striking signature was provided by the annexin family, with ANXA1, ANXA2, ANXA3, ANXA4, ANXA5, and ANXA11 all displaying compartment-dependent gradients. Together with the related Ca^2+^-binding proteins S100A6 and S100A10, these annexins highlight spatial variation in membrane trafficking, vesicle-associated processes, and Ca^2+^-regulated signaling—biology directly relevant to hormone exocytosis in islets.

Together, this analysis demonstrates that proteins with reversed spatial abundance gradients between islet and acinar compartments are strongly enriched in structural, extracellular matrix, membrane-associated, and trafficking-related processes. Notably, these include shared molecular machineries (e.g., annexins, cytoskeletal regulators) that appear to be ‘repurposed’ in opposing spatial orientations across the two microenvironments, as well as proteins such as CD63 and RBP1 highlighted in **Supplementary Figure 5**, whose compartment-reversed patterns point to fundamentally different functional roles in endocrine versus exocrine tissue. These findings suggest that the pancreatic microenvironment is organized not only along the classical endocrine–exocrine axis, but also by gradual, compartment-specific protein abundance gradients reflecting local differences in tissue architecture, cell–matrix interaction, and membrane dynamics. This level of organization as revealed by 3D spatial compartment-aware correlation analysis provides a new conceptual framework for interpreting how molecular programs are spatially organized within heterogeneous tissues, with implications for understanding diseases such as diabetes where these microenvironmental gradients are likely disrupted.

### Cell type deconvolution to create pseudo-single-cell-resolution images

While our spatial proteomics analysis offered new insights into the spatial context of the human islet microenvironment in 3D manner, the 50 μm pixel resolution of nanoPOTS-LCM does not achieve true single-cell resolution, limiting the ability to resolve molecular heterogeneity within mixed pixel cell populations. To address this limitation, we develop a workflow that integrates cell segmentation with protein intensity remapping by coupling confocal IF imaging with scRNA-Seq–derived correction factors. This strategy enabled the assignment of proteomic abundance profiles to individual cell types at single-cell resolution.

**Fig. 6 |:**
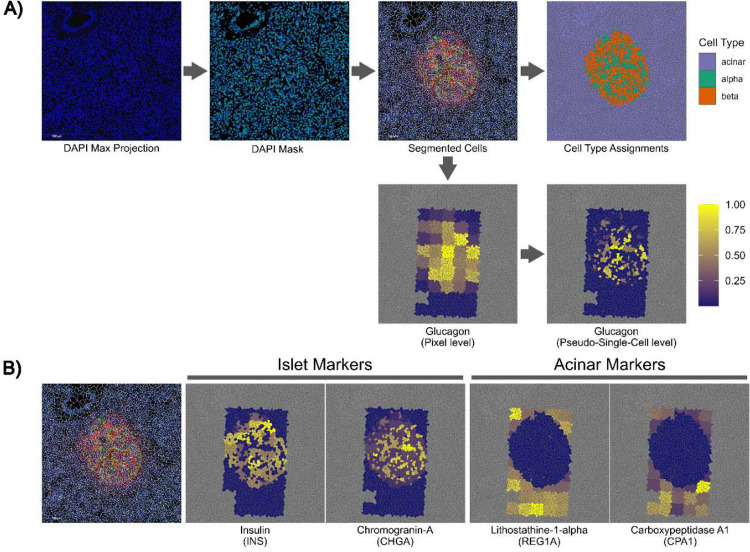
Cell type deconvolution of spatial proteomics data using immunofluorescence-guided segmentation. **a** Workflow of deconvolution procedure to obtain pseudo single-cell resolution images. DAPI staining was used to segment nuclei from adjacent tissue sections, and nuclear boundaries were defined using Cellpose 2.0. Cells were segmented by applying the Watershed algorithm to the identified nuclei. Segmented cells were classified by merging immunofluorescence data from DAPI, INS, and GCG channels, with cell types assigned based on dominant fluorescence: beta cells (red/INS), alpha cells (green/GCG), and acinar cells (blue/DAPI). These cell masks were linked to spatial proteomic pixels to associate protein intensities with individual cells. Cell type specific correction factors were applied to provide pseudo-single-cell resolution protein maps. **b** Spatial localization of representative pancreatic markers visualized at pseudo-single cell level. Heatmaps show protein intensities for individual cell types, highlighting spatially distinct molecular patterns within the pancreatic microenvironment.

Nuclei were first segmented using DAPI-stained tissue sections and processed to generate DAPI masks that defined nuclear boundaries. Cells were segmented using the Watershed algorithm applied to the DAPI mask, and cell types were assigned using the IF channels for INS, GCG, and DAPI. Cells were classified as beta cells (INS-high, red), alpha cells (GCG-high, green), or acinar cells (DAPI-dominant, blue) based on their RGB fluorescence intensity profiles. These cell segmentation masks were then aligned with the 50 μm spatial proteomic pixels, associating each segmented cell with pixel-level proteomics data (Figure 6a). To improve the resolution and achieve pseudo-single-cell-resolution proteomic maps, we applied cell type-specific correction factors derived from scRNA-Seq data from the Azimuth pancreas reference dataset^36, 37, 38, 39, 40, 50, 51^. This correction step deconvolved pixel-level protein intensities into refined, cell-type-resolved abundance estimates, enabling more accurate attribution of protein signal to its likely cellular source.

The resulting pseudo-single-cell proteomic maps recapitulated expected pancreatic cell-type biology (Figure 6). Endocrine islet markers such as INS and chromogranin-A (CHGA) were spatially localized within islets (Figure 6). Similarly, exocrine acinar markers such as Lithostathine-1-alpha (REG1A) and Carboxypeptidase A1 (CPA1) were enriched in acinar regions. Together, these results demonstrate that integrating IF-based cell segmentation, pixel-level proteomics, and scRNA-Seq–derived correction factors yields cell-type-specific molecular landscapes that capture the spatial and functional distinctions between endocrine (INS, CHGA) and exocrine (REG1A, CPA1) compartments. By bridging the resolution gap inherent to pixel-based workflows, this pseudo-single-cell strategy provides a generic framework for mapping single-cell proteome distributions within complex tissues by leveraging scRNAseq data.

### Interactive Web Portal for Visualization of 3D Spatial Proteomics and Immunofluorescence Data

**Fig. 7|:**
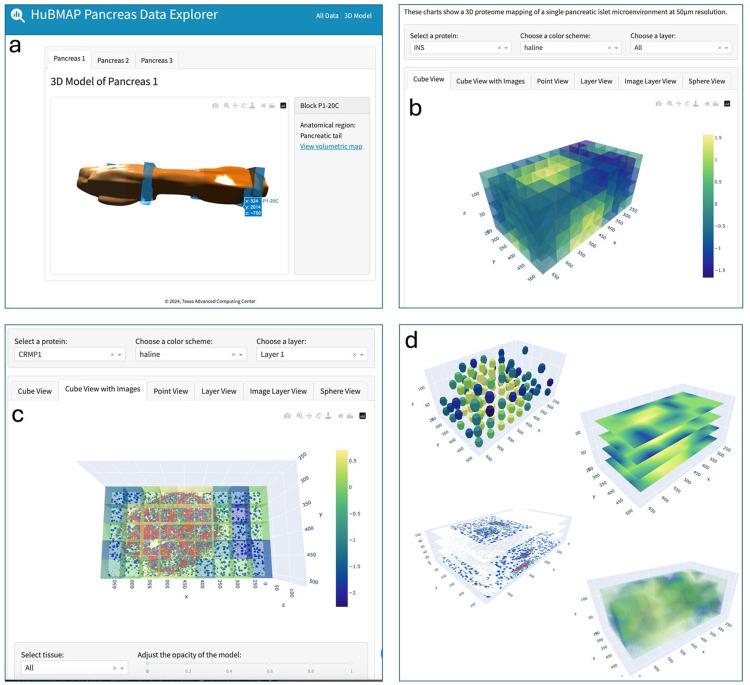
Interactive web-based access to the Spatial Proteome Data. **a** Block P1–20C which is the source of the volumetric proteome can be selected from the 3D view of the pancreas. **b** Users can select any of the proteins to view using transparent cubes. The volume can be rotated and individual layers selected. **c** Immunofluorescence images (see Figure 3a) can be viewed superimposed with the selected proteomic data in the volume cubes. **d** Multiple additional 3D viewing formats are supported for the proteomic and immunofluorescent images.

To enable intuitive exploration and visualization of our extensive 3D spatial proteomics data set, we developed an interactive, web-based viewer that allows researchers to access, query, and visualize proteins of interest in a user-friendly format (Figure 7). The platform was made through a custom implementation of an open-source visualization framework^52^, providing a flexible and scalable interface for multi-modal data dissemination.

As its core, the viewer presents the 50 μm resolution spatial proteome map in 3D, enabling detailed visualization of protein intensities across pixels and tissue sections. In parallel, the platform displays the corresponding confocal IF images for DAPI, INS, and GCG, allowing users to examine the high-resolution distribution of alpha and beta cells within the islets. By overlaying these complementary data modalities, the viewer enables direct correlation of spatial proteomic features with endocrine cell distributions, offering a comprehensive 3D view of the islet microenvironment.

The platform is publicly accessible at https://pancreas.tacc.utexas.edu, ensuring broad availability of this high-resolution dataset to the research community. As part of the HuBMAP consortium, the viewer serves as a vehicle for sharing integrated spatial datasets, contributing to the consortium’s mission of generating a comprehensive molecular and cellular atlas of human tissues.

## Discussion

In this study, we present a 3D spatial proteomics map of the human islet microenvironment at 50 μm resolution, achieved through the integration of IF imaging, LCM, and a nanoPOTS-LC-MS/MS workflow. By coupling this experimental pipeline with advanced computational analyses—including PCA, unsupervised Leiden clustering, and 3D spatial correlation mapping—our work provides new insights into the molecular gradients, transitional zones, and cell type–specific proteomic landscapes that define the endocrine–exocrine architecture of the human pancreas.

While prior spatial proteomics studies have primarily classified binary tissue components (e.g., islet vs. acinar)^24^, our unsupervised clustering approach uncovered four molecularly distinct microenvironments representing a continuum from acinar-dominant (Cluster 1), through ductal–stromal (Cluster 2), to islet-mantle (Cluster 3) and islet-core (Cluster 4) regions. Importantly, Cluster 2 revealed a peri-islet ductal–stromal niche enriched in canonical collagens (COL1A1, COL1A2, COL3A1) together with emerging ECM-associated proteins (ANXA4, SAFB2, KRT8/15/19), nominating it as a fibrotic-prone microenvironment relevant to diabetes^53, 54^. Likewise, Clusters 3 and 4 captured an islet core-to-mantle proteomic gradient, a zonation rarely resolved by conventional bulk approaches. Beyond canonical collagens, our analysis nominated several novel candidate ECM-associated proteins distributed across compartments: GP2 in the acinar zone (whose uromodulin-like ZP domain architecture suggests fibrillar scaffold activity), ANXA4 and SAFB2 in the ductal–stromal niche, and TGM2 and CLU in the islet niche. TGM2’s well-established role as an extracellular matrix crosslinker, together with CLU’s matricellular activity, suggests previously underappreciated mechanisms of islet-intrinsic ECM remodeling that may contribute to peri-islet fibrosis in type 2 diabetes.

Our 3D spatial correlation analysis further demonstrates the value of mapping of protein abundances against the geometric distance from the islet center. Glucagon (GCG) displayed a strong negative correlation with distance (R = −0.862), consistent with alpha-cell localization at the islet periphery^45, 46, 55^, while phospholipase A2 group IB (PLA2G1B) showed a strong positive correlation (R = 0.82), reflecting its role in lipid digestion within peripheral acinar regions (Supplementary Figure 5).

A particularly interesting finding enabled by our 3D spatial framework is the identification of a class of proteins displaying reversed abundance gradients between islet and acinar compartments (R2 × R3 < 0). Unlike proteins with monotonic gradients across the tissue (e.g., GCG and PLA2G1B), these reversed-gradient proteins exhibit diametrically opposed spatial behaviors depending on local cellular context, suggesting that shared molecular machineries are functionally repurposed across the endocrine–exocrine boundary—a phenomenon invisible to bulk or 2D approaches and uncovered here only by correlating protein abundances to a defined geometric reference (the 3D distance from the islet center). Two representative examples illustrate the biological significance of this pattern (Supplementary Figure 5). RBP1 displayed a positive correlation in islet pixels but a negative correlation in acinar pixels, consistent with the compartment-specific roles of retinoid signaling in supporting beta-cell identity within islets while regulating pancreatic stellate cell quiescence in the surrounding exocrine tissue—loss of which is a hallmark of fibrotic activation^56, 57^. Conversely, Conversely, CD63, a tetraspanin central to exosome and late-endosome biogenesis, displayed the opposite pattern—negative correlation in islet pixels, positive in acinar pixels—consistent with concentrated exosome-mediated signaling within the islet core and vesicular zymogen trafficking in acinar tissue^58^. Beyond these, the reversed-gradient protein set was enriched in extracellular matrix organization and fibrosis, cytoskeletal and membrane–cortex coupling, intermediate filament networks, and annexin- and Ca^2+^-mediated membrane trafficking. Collectively, these categories represent the structural, secretory, and signaling backbone of pancreatic tissue, suggesting that the islet–exocrine interface is shaped not only by cell-type-specific markers but also by compartment-specific organization of cellular machineries.

This finding has several implications. It supports the view that the peri-islet region is a biologically distinct microenvironment rather than a passive boundary, provides a framework for interpreting disease-associated remodeling in diabetes, pancreatitis, and pancreatic cancer. Importantly, the stratified correlation strategy is generalizable to any tissue microenvironment with definable architectural landmarks (e.g., glomerulus–tubule axis), offering an approach for uncovering microenvironment-specific functional gradients in heterogeneous tissues.

While our integrated 3D spatial proteomics workflow provides valuable new insights into the human pancreatic islet microenvironment, several limitations of this study should be acknowledged. First, the 50 μm pixel resolution of nanoPOTS-LCM proteomics, although enabling deep proteome coverage, does not achieve true single-cell resolution and inevitably averages protein signals across multiple cells within each pixel. Although we attempted to partially address this through pseudo-single-cell deconvolution workflow combining IF-based cell segmentation with scRNAseq–derived correction factors, this approach remains an inference-based deconvolution rather than direct single-cell measurement. Second, the current work only mapped one single islet microenvironment due to the proof-of-principle nature of the study as well as the limited throughput of the overall workflow. Future work will involve continual optimization and automation of the workflow to allow higher throughput mapping of islet microenvironments in both control and patient tissues. Third, the IF panel was restricted to DAPI, INS, and GCG, limiting cell-type assignment primarily to beta, alpha, and broadly defined acinar cells; minor cell populations such as delta, gamma, ductal, endothelial, and immune cells could not be directly resolved by IF.

In conclusion, the spatial proteomics workflow presented here establishes a robust framework for investigating tissue organization and molecular gradients in human pancreatic tissue. By combining nanoPOTS-LCM proteomics, 3D spatial correlation analysis, unsupervised clustering, and pseudo-single-cell segmentation, this work reveals biological insights into the islet microenvironment and a generalizable methodological foundation. We anticipate that this approach will pave the way for future applications across heterogeneous tissues, accelerating disease research, biomarker discovery, and multi-omics integration in the rapidly evolving spatial omics field.

While this study highlights the use of spatial proteomics, future endeavors could expand by integrating multi-omics approaches, including spatial transcriptomics^59, 60, 61^, spatial epigenomics such as DBiT-seq to explore chromatin modifications^62^ and advanced imaging platforms like multiplexed imaging methods (e.g., CODEX) to capture cellular and molecular contexts at high spatial resolution^63^. These technologies, combined with spatial proteomics, could provide a more holistic molecular characterization of pancreatic tissues and enable cross-modal validation of the gradients and ECM signatures identified here.

## Methods

### Human pancreas tissue collection

Human pancreas tissue was acquired from a 17-year-old male donor. Selection of the donor was guided by eligibility criteria set forth by the HuBMAP consortium, adhering to protocol IRB201600029 (https://www.protocols.io/view/donor-eligibility-criteria-and-pancreas-recovery-f-b7nfrmbn)^25^, along with the ethical guidelines outlined in the Declaration of Helsinki. The recovery and processing of the organ occurred at the University of Florida, strictly following an established protocol (https://www.protocols.io/view/human-pancreas-processing-b7gxrjxn)^26^. Tissue block embedded in 2.6% carboxymethylcellulose (CMC) media was frozen with dry ice/isopentane bath and stored in −80 °C freezer.

### Immunofluorescence staining

The tissue slide was fixed in 4% paraformaldehyde for 10 mins, followed by three washes with phosphate buffered saline (PBS). Tissue section was permeabilized in 0.1% Triton X-100 in phosphate buffered saline for 10 mins and blocked with 1% BSA in PBST (PBS with 0.05% Tween20) for overnight at 4 °C. The tissue section was incubated with three antibodies at 1:100 dilution; BD Pharmingen^™^ PE Mouse Anti-Glucagon (BD bioscience, cat# 565860) and BD Pharmingen^™^ Alexa Fluor^®^ 647 Mouse Anti-Insulin (BD bioscience, cat# 565689) in 1% BSA in PBST at 4 °C overnight. The slide was washed three times with ice-cold PBS followed by incubating with Alexa Flour 488 goat anti-rabbit IgG (abcam Cat# ab150077) at 1:1000 dilution in 1× PBS with 1% BSA for an hour at room temperature in the dark.

### Confocal Imaging

Prior to image scanning, the slides were treated with a 1 μg/mL DAPI solution in PBS for 15 minutes at room temperature, followed by a wash with PBS. Next, 20 μL of VECTASHIELD^®^ antifade mounting medium (Vectorlabs) was applied to the tissue, and a glass coverslip was carefully placed on top. The edges of the coverslip were then sealed using nail polish. Imaging was conducted using a Zeiss LSM 710 confocal microscope. For whole slide scans, a 10X NA 0.3 objective was employed, while a 40X NA 1.1 water immersion objective was used to achieve high-resolution images of target islet. The appropriate fluorophores were excited using lasers at wavelengths of 405 nm, 488 nm, 561 nm, and 633 nm.

### NanoPOTS chip fabrication

Nanowell chips were fabricated using the established design and fabrication techniques described previously^64^. Briefly, a grid of 4 × 11 nanowells was created on glass slides precoated with chromium and photoresist (dimensions 25 mm × 75 mm, provided by Telic company). Using photolithography and wet etching techniques, nanowells measuring 1.2 mm in a diameter were fabricated, spaced 4.5 mm apart from center to center. To ensure proper surface properties, the exposed glass surfaces were treated with 2% (v/v) heptadecafluoro-1,1,2,2-tetrahydrodecyldimethylchlorosilane in 2,2,4-trimethylpentane. Finally, the residual chromium layer was removed, leaving hydrophilic spots in nanowells for tissue sample collection and proteomic processing.

### Laser capture microdissection

Pancreatic tissue sections (10 μm thick) were collected on PEN membrane slides (Carl Zeiss) and stained using an H&E staining kit (Abcam) following the manufacturer’s instructions. To optimize sample capture, each nanowell was preloaded with 200 nl dimethyl sulfoxide (DMSO)^29^. Laser capture microdissection (LCM) was performed on a PALM MicroBeam LCM system (Carl Zeiss MicroImaging), with pixelation achieved by drawing a gridline around the region of interest followed by tissue cutting and catapulting. Tissue was cut at an energy level of 40 using the “CenterRoboLPC” function of PalmRobo software. For catapulting, an energy level of delta 15 and a focus level of delta 5 were used to transfer tissue pixels into nanowells. After capture, the chip was incubated at 70 °C for 20 minutes under hanging drop mode to evaporate the DMSO droplets^65^. Successful tissue collection was confirmed via microscopy, and chips were stored at −20 °C until used for downstream analysis.

### NanoPOTS-Based Proteomic Sample Processing

Proteomic sample processing followed a nanoPOTS workflow adapted from previous studies^28, 66^. A home-built nanoliter robotic platform was used for reagent delivery into nanowells. First, 200 nl lysis buffer containing 0.1% (w/v) n-dodecyl-β-D-maltoside, 1 mM tris(2-carboxyethyl)phosphine, and 0.1 M HEPES (pH 8.0) was added per well and incubated at 70°C for 1 hour. Alkylation was achieved by adding 50 nl of 10 mM 2-chloroacetamide, followed by incubation at room temperature for 30 minutes. Protein digestion was performed overnight at 37 °C with 50 nl of 0.01 ng nl^−1^ Lys-C (MS grade, Promega) and 50 nl of 0.04 ng nl^−1^ trypsin (Promega) in 0.1 M HEPES buffer, placing the chip upside down to enhance lysis and digestion efficiency^65^. Digestion was quenched with 50 nl 5% formic acid and dried under vacuum before storage at −20 °C.

### LC-MS/MS analysis

LC-MS/MS was conducted using a homebuilt nanoPOTS autosampler equipped with a in-house packed solid-phase extraction column (100 μm i.d., 4 cm, packed with 5 μm, C18 packing material (300 Å pore size; Phenomenex), and an in-house packed LC column (50 μm i.d., 25 cm-long, packed with 1.7 μm, C18 packing material (BEH 130 Å C18 material, Waters) heated at 50 °C with a column heater (Analytical Sales and Services Inc). Dried peptide on nanowell were reconstituted with buffer A (0.1% formic acid in water), and then loaded into the solid-phase extraction column for 5 min with a buffer A at 3 μl min^−1^. Peptide separation was performed using an UltiMate 3000 RSLCnano System (Thermo Fisher Scientific) and an Orbitrap Lumos Tribrid MS (Thermo Fisher Scientific) with a field asymmetric waveform ion mobility spectrometry (FAIMS) pro interface. A 30 min linear gradient from 8% to 22% buffer B (0.1% formic acid in acetronitrile) followed 9 min linear gradient from 22% to 35% buffer B at 100 nl min^−1^. Ionization was performed at the electrospray source (ESI) with a 2400 V.

For spectrum library construction, ionized peptides were fractionated by the FAIMS Pro interface, using discrete compensation voltages of −45, −60, or −75 V during each LC-MS run. Fractionated ions were transferred into a heated ion transfer tube (200 °C) and scanned at a resolution of 120,000 across a mass range of 350–1600 *m/z*, with an injection time of 118 ms and an automatic gain control (AGC) target set to 1E6. Precursor ions with charge states of +2 to +6 and intensities greater than 1E4 were isolated with a 1.4 *m/z* window and fragmented using high-energy dissociation (HCD) set at 30%. Fragmented ions were scanned in an ion trap with a maximum injection time of 100 ms and an AGC target of 2E4, with a total cycle time of 2 s.

For the single-pixel analysis, ionized peptides were fractionated using the FAIMS Pro interface across three compensation voltages (−45, −60, −75 V) for each LC-MS experiment. The full MS was performed at 120,000 resolution, with an injection time of 245 ms and an AGC target of 1E6. Precursor ions with charges from +2 to +6 and intensities greater than 1E4 were isolated within a 1.4 *m/z* window and fragmented via HCD at 30%. Fragmented peptide ions were scanned in an ion trap with an injection time of 86 ms and an AGC target of 2E4, with the cycle time for each compensation voltage experiment set to 0.8 s.

### MS Data analysis

All spectrum raw files were processed using FragPipe (v 18.0), powered by MSFragger (v. 3.5)^67, 68^ search engine, with Philosopher (ver. 4.4.0)^69^ for false discovery rate (FDR) filtering and reporting, and IonQuant (v 1.8.0)^70^ for label-free quantification (LFQ) and match-between-runs (MBR) analysis. All mass spectrum was searched against a UniProt human (Homo sapiens) database (April 2, 2022 release), which included 20,318 protein sequences, 116 common contaminant sequences, and decoy protein sequences. For MS/MS analysis, search parameters included full tryptic specificity, allowing for up to two missed cleavage sites. Carbamidomethylation (+57.0214 Da) on cysteine was specified as a fixed modification, while methionine oxidation (+15.9949 Da) and protein N-terminal acetylation (+42.0105 Da) were set as variable modifications. Precursor and fragment mass tolerances were initially set to 20 ppm, and automatic calibration by MSFragger adjusted these tolerances during processing. Identification results were filtered at both the peptide-spectrum match (PSM) and protein levels with a 1% FDR threshold.

Quantification utilized the match-between-runs algorithm within IonQuant with a retention time (RT) matching tolerance of 0.4 min, an *m/z* tolerance of 10 ppm, and an ion-level FDR threshold of 5%. Protein-level intensities were normalized and computed using the MaxLFQ algorithm.

### Spatial proteomics data analysis

Statistical analyses were conducted using R (v4.3.2; https://www.r-project.org) and Perseus (v1.6.15.0; https://maxquant.net/perseus/). Protein abundance columns were extracted from the “combined_protein.tsv” report files and log2 transformed. For further visualizations and downstream analyses, only proteins with over 70% valid values across the 177 tissue pixels were considered quantifiable. Missing values were imputed based on the standard distribution of valid values (width: 0.3, downshift: 1.8) within Perseus.

Spatial proteomics analyses were conducted using the Giotto package, with default embedded parameters. The raw protein abundance matrix obtained from FragPipe, along with spatial pixel coordinates, was used to create a Giotto object. After normalization, Leiden clustering was applied to detect spatial protein groups, which were visualized using Principal Component Analysis (PCA) plots to explore molecular distinctions among tissue regions.

For pathway and cell-type enrichment analyses, Enrichr (https://maayanlab.cloud/Enrichr/) was used. Proteins from each Leiden cluster were uploaded to Enrichr for Reactome pathway enrichment and cell-type marker analyses. Enrichment results, including pathway and cell-type profiles, were exported for further interpretation to uncover biological features associated with tissue compartments.

### Pseudo-single cell proteomics deconvolution

Pseudo-single cell-level proteomics deconvolution was performed by combining spatial proteomics data with single-cell segmentation guided by nuclei localization from DAPI-stained adjacent serial tissue section. The DAPI maximum projection was submitted to the Cellpose 2.0^71^ software for identification of location and boundary of nuclei. The resulting digital nuclei mask was used to perform cell segmentation in ImageJ (Fiji)^72^ with the Watershed algorithm and particle analysis to provide individually addressable cell areas. Cell boundary coordinate files were imported into R using the RImageJROI package (https://CRAN.R-project.org/package=RImageJROI) and converted to polygons with the sf package (https://CRAN.R-project.org/package=sf). Cell type assignment was performed by merging immunofluorescence images of DAPI, insulin, and glucagon channels and quantifying the mean RGB values of each cell using ImageJ with python automation. The dominant color in each cell area was used to assign cell type. Red-dominant cells were assigned as beta cells (INS-high), green-dominant cells were assigned as alpha cells (GCG-high), and blue-dominant cells were assigned to be acinar cells. After cell type assignment, cells were mapped to their corresponding nanoPOTS tissue pixels by overlaying the segmented cell regions of interest (ROI) with a nanoPOTS pixel mask representing the locations of the spatial proteomics. Each pixel was represented by a distinct color, and the dominant color value within each cell ROI was measured in ImageJ and exported for processing in R. This allowed for the assignment of cell ROIs to specific pixels, effectively linking cell segmentation to spatial proteomic data. Protein abundance values from pixel-level proteomics were assigned to their corresponding cells, enabling pseudo-single-cell-level proteomic resolution. To refine the protein intensities for each cell type, a correction factor was applied based on single-cell RNA sequencing (scRNA-Seq) data from the Azimuth pancreas reference dataset.^36, 37, 38, 39, 40, 50, 51^. The correction factor was calculated for each gene as the ratio of counts observed in alpha, beta, and acinar cells, relative to the combined counts for these cell types.

### Web-based viewer construction

Tissue Data Explorer is written in Python with Plotly Dash, which provides a Flask server and wraps React components in Python as described in Labyer 2026^52^. The https://pancreas.tacc.utexas.edu server is hosted on Jetstream 2^73^.

## Supplementary Material

Supplementary Files

This is a list of supplementary files associated with this preprint. Click to download.

- SItable1tissuesectionuse.xlsx
- SItable4immunofluorescenceresult.xlsx
- SItable5pixelmetadata.xlsx
- SItable6Azimuthcelltypemarker.xlsx
- SItable7DistanceCorrelation.xlsx
- SItable3proteomeresult.xlsx
- SupplementaryInformation.docx
- SItable2LCMpixelation.xlsx

The supplementary information includes 5 supplementary figures and 7 supplementary tables.

## Data Availability

The MS raw data can be accessed on the ProteomXchange Consortium via the MassIVE partner repository with the data set identifier MSV000102174 and are available at ftp://MSV000102174@massive-ftp.ucsd.edu (Password for reviewer: Map4890)
